# Association Study of *SLCO1B3* and *ABCC3* Genetic Variants in Gallstone Disease

**DOI:** 10.3390/genes13030512

**Published:** 2022-03-14

**Authors:** Bolesław Banach, Andrzej Modrzejewski, Zygmunt Juzyszyn, Mateusz Kurzawski, Tomasz Sroczynski, Andrzej Pawlik

**Affiliations:** 1 Department of Physiology, Pomeranian Medical University in Szczecin, 70-111 Szczecin, Poland; banbol@pum.edu.pl (B.B.); zjuzyszyn@wp.pl (Z.J.); tomasz.sroczynski@pum.edu.pl (T.S.); 2 Clinical Department of General Surgery, Pomeranian Medical University in Szczecin, 70-111 Szczecin, Poland; andrzej.modrzejewski@pum.edu.pl; 3 Department of Experimental and Clinical Pharmacology, Pomeranian Medical University, 70-111 Szczecin, Poland; mateusz.kurzawski@pum.edu.pl

**Keywords:** gallbladder disease, *SLCO1B3*, *ABCC3*, genetic polymorphism, bile acids

## Abstract

There is growing evidence that gallstone formation may be genetically determined. Recent studies have shown that polymorphism of genes encoding proteins involved in bile acid transport may be associated with the risk of gallstone disease. The aim of this study was to investigate the association between *SLCO1B3* (rs4149117:G>T, rs7311358:A>G) and *ABCC3* (rs4793665:T>C, rs11568591:G>A) genetic variants and susceptibility to cholesterol gallstone disease, as well as gallstone composition. The study included 317 patients suffering from cholelithiasis who underwent cholecystostomy and 249 controls with no evidence of stones, confirmed by ultrasound examination. There were no statistically significant differences in the distribution of studied gene polymorphisms between patients with gallstone disease and healthy controls. No significant associations were observed between studied genotypes and the content of analyzed gallstone components: total cholesterol, bilirubin, CaCO_3_, nor the total bile acids. There was also no association between bile acid content in gallstones and the polymorphisms studied. The results of this study suggest that polymorphisms of *SLCO1B3* and *ABCC3* genes are not a valuable marker of gallstone disease susceptibility and do not influence gallstone composition.

## 1. Introduction

Cholelithiasis is one of the most common gastroenterological illnesses. In Europe and the United States, it affects 10 to over 20% of the population [1]. There are many identified environmental risk factors for gallstone disease (GSD). The most important are: age, gender, diet, obesity, hyperlipidemia, diabetes, elevated estrogen levels, pregnancy, liver cirrhosis and hemolytic disease [2]. The role of genetic factors in the disease pathogenesis is widely discussed. Observations proving the occurrence of GSD in humans of different geographic regions, different ethnic groups, within families, and in monozygous and heterozygous twins directly indicate the presence of genetic conditions. The contribution of genetic factors in the pathogenesis of GSD is estimated to be from about 20% to over 40% [3]. Monogenic predisposition has been described in carriers of rare mutations in phosphatidylcholine transporter (*ABCB4*) and cholesterol transporter (*ABCB11*) genes [4,5], and more recently, a common gene polymorphism in *ABCG8* was associated with cholesterol gallstone disease [6].

The primary cause of gallstone disease is cholesterol oversaturation of the bile, induced by hypersecretion of biliary cholesterol or decreased secretion of bile salts [7,8]. Hepatic uptake of bile acids is mediated by high-affinity Na^+^-dependent bile salt transporter (NTCP, encoded by *SLC10A1*) and a family of multispecific organic anion transporters (OATPs) [9]. In humans, three liver-specific OATPs expressed in basolateral membrane of hepatocytes, OATP1A2 (encoded by *SLCO1A2*), OATP1B1 (*SLCO1B1*) and OATP1B3 (*SLCO1B3*), are responsible for Na^+^-independent uptake of bile acids. In addition to the uptake system, the basolateral membrane also contains efflux pumps, MRP3 (*ABCC3)* and MRP4 (*ABCC4*), responsible for ATP-dependent efflux of biliary constituents into portal blood. The expression of MRPs is normally observed in hepatocytes at a very low level, but is significantly up-regulated in cholestasis [10,11]. Among those transporters, *SLCO1B3* and *ABCC3* revealed functional genetic polymorphism, influencing protein expression, and potentially affecting the hepatic uptake of bile salts. Two common single-nucleotide polymorphisms (SNPs) within *SLCO1B3*, affecting the OATP1B3 amino acid sequence, were described: rs4149117:G>T in exon 3, resulting in 112Ser>Ala change, and rs7311358:A>G in exon 6, leading to 233Met>Ile amino acid substitution. Both SNPs are in high linkage in Caucasians, i.e., two missense polymorphisms produce a variant haplotype [12]. Polymorphic alleles of *ABCC3* were shown to influence the transport rate of some, but not all, OATP1B3 substrates [13,14,15]. A common promoter polymorphism was described in *ABCC3* gene (rs4793665:T>C in position −211) and associated with altered hepatic MRP3 mRNA expression [16], whereas among SNPs altering amino acid sequence, rs11568591:G>A in exon 27 (1297Arg>His) appears with relatively high frequency in Caucasians [17].

The aim of this study was to investigate the association between *SLCO1B3* (rs4149117:G>T, rs7311358:A>G) and *ABCC3* (rs4793665:T>C, rs11568591:G>A) genetic variants and susceptibility to cholesterol gallstone disease, as well as gallstone composition.

## 2. Patients and Methods

### 2.1. Patients

This study included 317 patients (232 women and 85 men) diagnosed with cholelithiasis, who underwent cholecystectomy in years 2018–2019 at the Department of Surgery, Pomeranian Medical University, Szczecin, Poland, and 249 healthy individuals (131 women, 118 men) with no evidence of gallstones, confirmed by ultrasound examination, as the control group. The stones were proven to be cholesterol-type gallstones by chemical analysis, showing cholesterol concentration over 70%. The study was approved by the ethics committee at Pomeranian Medical University, Szczecin, Poland (KB-0009/18/07), and written informed consent was obtained from all subjects.

### 2.2. Genotyping

Patients were genotyped for the presence of two functional SNPs in *ABCC3* gene (rs4793665:C>T in position −211 of promoter sequence, and rs11568591:G>A in exon 27, resulting in 1297Arg>His amino acid substitution) and two SNPs in *SLCO1B3* (rs4149117:T>G in exon 3 −112Ser>Ala, and rs7311358:G>A in exon 6 −233Met>Ile). Genomic DNA was extracted from 200 µL of whole-blood samples using GeneMATRIX Quick Blood DNA Purification Kit (EURx, Gdańsk, Poland) (catalog number: E3565-02). The allelic discrimination TaqMan real-time PCR assays (Assay IDs: C__27829307_10, C__31810858_20, C__25639181_40, C__25765587_40) (Applied Biosystems, Bedford, MA, USA) (catalog numbers: 4351376 and 4362691) were used for detection of the studied SNPs. Fluorescence data were captured using an ABI PRISM 7500 FAST Real-Time PCR System (Applied Biosystems, Bedford, MA, USA), after 40 cycles of PCR.

### 2.3. Analysis of Gallstone Composition

During cholecystectomy, gallstones were collected from the gall bladder for analysis. The gallstones were washed with distilled water, dried in a desiccator and powdered with a mortar. Two probes (100 mg) of each stone were dissolved in 10 mL of DMSO with tert-butyl ester (8:2). Extraction was conducted for 24 h in a thermostated shaker (25 °C). The following analyses were performed on the obtained extract: total bile acids (enzymatic kit, Merck), Darmstadt, Germany, bilirubin and total cholesterol (Biosystem diagnostic tests) and bile acid composition. Bile acids were analyzed by high-pressure liquid chromatography (HPLC) with UV-VIS detector. The following bile acids were analyzed: cholic acid (CA), chenodeoxycholic acid (CDCA), taurocholic acid (TCA), lithocholic acid (LCA), glycochenodeoxycholic acid (GCDCA), glycocholic acid (GCA), and taurochenodeoxycholic acid (TCDCA).

### 2.4. Statistical Analysis

The consistency of the genotype distribution with Hardy–Weinberg equilibrium (HWE) was assessed using the Fisher exact test. The genotype and allele distributions were compared between groups using the chi-squared test and the Fisher exact test. The distribution of quantitative parameters of gallstone components differed significantly from the normal distribution (Shapiro–Wilk test), so they were compared between groups using the non-parametric Kruskal–Wallis test and the Mann–Whitney test, *p* < 0.05 was considered statistically significant. Statistical analysis was performed using Statistica 13.3 software.

## 3. Results

Clinical characteristics of patients and control subjects are shown in Table 1. The distribution of all analyzed SNPs, i.e., rs4793665:C>T and rs11568591:G>A in *ABCC3* as well as rs4149117:T>G and rs7311358:G>A in *SLCO1B3*, was in concordance with Hardy-Weinberg equilibrium. There were no statistically significant differences in the distribution of studied gene polymorphisms between patients with gallstone disease and healthy controls (Table 2).

The composition of gallstones was analyzed in 51 patients. The components of gallstones were compared between the *ABCC3* and *SLCO1B3* genotypes. There were no statistically significant differences in the analyzed gallstone components of total cholesterol, bilirubin, CaCO_3_, or total bile acids between patients with different *ABCC3* and *SLCO1B3* genotypes (Table 3, Table 4 and Table 5). There was also no association between bile acid content in gallstones and the polymorphisms studied (Table 3, Table 4 and Table 5).

There was a total linkage between rs7311358:G>A and rs4149117:T>G in this subgroup of patients.

## 4. Discussion

Recent studies have shown that polymorphism of genes encoding proteins involved in bile acid transport may be associated with the risk of gallstone disease [6,18,19]. Hence, we decided to analyze in that context the potential role of functional polymorphism in two genes expressed in basolateral membrane of hepatocytes: *SLCO1B3* (one of the proteins responsible for Na^+^-independent uptake of bile acids) and *ABCC3* (involved in ATP-dependent bile acids efflux). However, our results show that there is no association between the studied SNPs and GSD. It can be expected that the functional polymorphisms of the transporters studied may affect their substrates, i.e., the bile acid content in the stones. However, substantial variability in gallstone composition between individuals observed in the current study was not related to *SLCO1B3* nor *ABCC3* genotypes. Negative results suggest that common polymorphisms in *SLCO1B3* and *ABCC3* genes do not influence the function of the encoded transporters to a large extent, and are not clinically important in the case of gallstone disease. A similar negative conclusion was drawn by Jindal et al. [20] in relation to *SLCO1B1* c.388G>A SNP that had been associated with altered plasma concentrations of bile acids [21].

The presence of polymorphisms associated with the risk of developing gallstone disease in humans has been demonstrated in genes encoding plasma transport and catabolism of cholesterol proteins (*APOE*, *APOB*, *APOA1*), and in the cholesterol esters transporter protein gene (*CEPT*). The *APOE* polymorphism is most frequently investigated in patients with gallstone disease [2,22,23].

Most data on the role of genetic factors in the cholesterol gallstone disease pathogenesis have been obtained in studies conducted on various strains of mice. The results of these studies were of significant help and inspiration in the search for analogous genes responsible for the formation of gallstones in humans. These studies demonstrated the association between cholesterol gallstone disease and the genes encoding liver regulatory enzymes (Hmgcr, Cypa1, Soat2), cholecystokinin receptor (Cckar), HDL receptor (Srb1), apolipoprotein (ApoE), basolateral organic cation transporter (Slc22a1), and tubular bile salt export pump (Abcb11) [24,25,26].

*ABCC3* gene rs4793665 polymorphism is located at position −211 in the promoter region of the gene. This variant results in decreased mRNA expression of the *ABCC3* gene due to reduced binding of transcription factors in the promoter region of the gene [27]. This polymorphism has been studied as a predisposing factor for acute myeloid and lymphoblastic leukemia and as a factor influencing the efficacy of therapy in these patients and in RA patients treated with methotrexate [27,28,29,30]. The *ABCC3* gene rs4793665 polymorphism was significantly associated with methotrexate pharmacokinetics both in juvenile idiopathic arthritis and in pediatric osteosarcoma patients [29,30].

The *SLCO1B3* gene rs4149117 and rs7311358 polymorphisms have been shown to affect the efficacy of statin treatment in patients with hypercholesterolemia. Additionally, these polymorphisms are associated with an increased risk for acute rejection and allograft failure in lung transplant recipients treated with mycophenolic acid [31,32]. To date, the above polymorphisms have not been investigated in relation to diseases of the liver and the gastrointestinal tract.

The results of our study showed no association between the polymorphisms studied and the risk of gallstone disease or the composition of gallstones. Gallstone disease is a complex disease that depends on many factors. Both genetic and environmental factors contribute to its development. In particular, diet, hormonal disorders—especially in the area of sex hormones—obesity, diabetes, lipid metabolism disorders, and medication, play an important role. The impact of genetic polymorphisms appears to be small; it must be considered together with other environmental risk factors.

We cannot exclude minor effects of *SLCO1B3* and *ABCC3* genetic polymorphisms on gallstone disease risk and gallstone composition, which could be masked by the variability of environmental factors within investigated patient populations.

## 5. Conclusions

The results suggest that common polymorphisms in *SLCO1B3* and *ABCC3* genes are not a valuable marker of gallstone disease susceptibility and do not influence gallstone composition.

## Figures and Tables

**Table 1:** Clinical characteristics of patients and control subjects.

Parameters	Patients	Control Group
	Mean ± SD	Mean ± SD
Age (years)	54.5 ± 15.2	62.6 ± 15.2
BMI (kg/m^2^)	26.4 ± 5.1	25.7 ± 4.8
CH (mg/dL)	216.3 ± 39.2	212 ± 37.8
HDL (mg/dL)	55.1 ± 13.3	57.4 ± 15.9
LDL (mg/dL)	119.2 ± 34.9	115 ± 34.2
TG (mg/dL)	140.2 ± 91.3	125 ± 78.5

**Table 2:** Frequency of *ABCC3* and *SCLCO1B3* alleles and genotypes in GSD patients and healthy controls.

	Gallstone Disease		Healthy Controls		*p* Value *	OR (95% CI)
	*n*	(%)	*n*	(%)		
	*n* = 317		*n* = 249			
*ABCC3* rs4793665:C>T						
TT	87	27.4%	76	30.5%	-	-
TC	167	52.7%	130	52.2%	0.558	1.12 (0.76–1.65)
CC	63	19.9%	43	17.3%	0.379	1.28 (0.78–2.10)
allele T	341	53.8%	282	56.6%	0.455	1.16 (0.81–1.67)
allele C	293	46.2%	216	43.4%		-
*ABCC3* rs11568591:G>A						
GG	293	92.4%	222	89.2%	-	-
GA	24	7.6%	26	10.4%	0.236	0.70 (0.39–1.25)
AA	0	0.0%	1	0.4%	0.432	-
allele G	317	93.0%	470	94.48%	0.186	0.67 (0.38–1.20)
allele A	24	7.0%	28	5.6%		-
*SLCO1B3* rs7311358:G>A						
AA	212	66.9%	160	64.3%	-	-
AG	94	29.6%	78	31.3%	0.642	0.91 (0.63–1.31)
GG	11	3.5%	11	4.4%	0.517	0.75 (0.32–1.78)
allele A	518	81.7%	398	79.9%	0.533	0.89 (0.63–1.26)
allele G	116	18.3%	100	20.1%		-
*SLCO1B3* rs4149117:T>G						
GG	212	66.9%	160	64.3%	-	-
GT	94	29.6%	79	31.7%	0.642	0.90 (0.62–1.29)
TT	11	3.5%	10	4.0%	0.821	0.83 (0.34–2.00)
allele G	518	81.7%	399	81.1%	0.533	0.89 (0.63–1.26)
allele T	116	18.3%	99	19.9%		-

* Fisher exact test.

**Table 3:** Composition of analyzed gallstones (% ± SD) in relation to *ABCC3* rs4793665 genotypes.

		rs4793665:C>T			*p* Value ^#^	*p* Value *		
		CC (*n* = 11)	CT (*n* = 26)	TT (*n* = 14)		*p*1	*p*2	*p*3
total cholesterol		79.7 ± 9.62	78.7 ± 12.55	78.4 ± 11.57	0.95	0.98	0.99	0.93
bilirubin		4.49 ± 3.85	5.98 ± 5.06	5.92 ± 4.75	0.62	0.55	0.46	0.86
CaCO_3_		8.09 ± 7.68	8.73 ± 7.76	7.99 ± 6.34	0.92	0.98	0.88	0.89
total bile acids		2.52 ± 2.16	1.94 ± 1.29	2.02 ± 1.42	0.81	0.73	0.59	0.86
Bile acids [% of total]	TCA	6.0 ± 8.2	6.3 ± 9.3	3.7 ± 8.1	0.29	0.20	0.38	0.23
	TCDCA	9.4 ± 9.4	13.1 ± 15.0	8.6 ± 11.5	0.47	0.34	0.63	0.34
	GCA	24.9 ± 22.2	25.8 ± 23.4	34.7 ± 35.3	0.81	0.89	0.52	0.99
	GCDCA	12.8 ± 23.3	14.2 ± 15.3	17.4 ± 17.8	0.23	0.20	0.21	0.24
	CA	5.9 ± 19.6	3.8 ± 11.3	5.4 ± 18.8	0.65	0.89	0.58	0.70
	CDCA	1.3 ± 2.3	8.1 ± 19.4	6.1 ± 9.6	0.48	0.37	0.42	0.57
	LCA	39.7 ± 39.8	28.7 ± 23.2	24.1 ± 19.1	0.37	0.32	0.26	0.51

*p* value ^#^—Kruskal–Wallis test, *p* value *—Mann–Whitney U-test; *p*1: CC vs. TT; *p*2: CC vs. CT + TT; *p*3: CC + CT vs. TT; abbreviations: cholic acid (CA), chenodeoxycholic acid (CDCA), taurocholic acid (TCA), lithocholic acid (LCA), glycochenodeoxycholic acid (GCDCA), glycocholic acid (GCA), and taurochenodeoxycholic acid (TCDCA).

**Table 4:** Composition of analyzed gallstones (% ± SD) in relation to *ABCC3* rs11568591 genotypes.

		rs11568591:G>A		
		GG (*n* = 46)	GA (*n* = 5)	*p* Value *
total cholesterol		79.3 ± 11.33	74.3 ± 13.67	0.345
bilirubin		5.27 ± 4.46	9.89 ± 5.71	0.091
CaCO_3_		8.52 ± 7.28	7.17 ± 7.59	0.611
total bile acids		2.14 ± 1.58	1.60 ± 0.95	0.841
Bile acids [% of total]	TCA	5.3 ± 8.5	8.1 ± 11.3	0.568
	TCDCA	10.7 ± 12.6	13.1 ± 13.6	0.987
	GCA	28.1 ± 26.5	26.4 ± 25.9	0.507
	GCDCA	15.2 ± 18.6	8.5 ± 9.2	0.377
	CA	5.1 ± 16.3	1.4 ± 3.1	0.938
	CDCA	5.8 ± 14.1	5.4 ± 8.7	0.938
	LCA	29.8 ± 29.7	37.1 ± 28.4	0.841

* Mann–Whitney U-test; abbreviations: cholic acid (CA), chenodeoxycholic acid (CDCA), taurocholic acid (TCA), lithocholic acid (LCA), glycochenodeoxycholic acid (GCDCA), glycocholic acid (GCA), and taurochenodeoxycholic acid (TCDCA).

**Table 5:** Composition of analyzed gallstones (% ± SD) in relation to *SLCO1B3* rs7311358:G>A genotypes.

		rs7311358:G>A		
		AA (*n* = 32)	AG + GG (*n* = 19)	*p* Value *
total cholesterol		78.5 ± 9.26	79.4 ± 9.52	1.000
bilirubin		5.48 ± 3.79	6.10 ± 4.23	0.404
CaCO_3_		8.55 ± 6.42	8.13 ± 6.28	0.992
total bile acids		2.26 ± 1.47	1.76 ± 1.32	0.824
Bile acids [% of total]	TCA	4.8 ± 8.1	7.2 ± 10.1	0.530
	TCDCA	8.1 ± 10.5	17.3 ± 16.6	0.112
	GCA	28.4 ± 26.6	26.9 ± 25.5	0.358
	GCDCA	14.2 ± 19.6	16.0 ± 15.0	0.411
	CA	6.6 ± 18.3	1.0 ± 2.3	0.595
	CDCA	6.5 ± 15.2	4.4 ± 9.4	0.422
	LCA	31.4 ± 30.3	28.6 ± 26.5	0.190

* Mann–Whitney U-test; abbreviations: cholic acid (CA), chenodeoxycholic acid (CDCA), taurocholic acid (TCA), lithocholic acid (LCA), glycochenodeoxycholic acid (GCDCA), glycocholic acid (GCA), and taurochenodeoxycholic acid (TCDCA).

## Data Availability

Not applicable.
